# Biomass Reallocation between Juveniles and Adults Mediates Food Web Stability by Distributing Energy Away from Strong Interactions

**DOI:** 10.1371/journal.pone.0170725

**Published:** 2017-01-23

**Authors:** Amanda L. Caskenette, Kevin S. McCann

**Affiliations:** Department of Integrative Biology, University of Guelph, Guelph, Ontario, Canada; Sveriges lantbruksuniversitet, SWEDEN

## Abstract

Ecological theory has uncovered dynamical differences between food web modules (i.e. low species food web configurations) with only species-level links and food web modules that include within-species links (e.g. non-feeding links between mature and immature individuals) and has argued that these differences ought to cause food web theory that includes within-species links to contrast with classical food web theory. It is unclear, however, if life-history will affect the observed connection between interaction strength and stability in species-level theory. We show that when the predator in a species-level food chain is split into juvenile and adult stages using a simple nested approach, stage-structure can mute potentially strong interactions through the transfer of biomass within a species. Within-species biomass transfer distributes energy away from strong interactions promoting increased system stability consistent with classical food web theory.

## Introduction

Ecosystem stability, the ability of an ecosystem to persist through time and resist perturbations, depends on the biological structure inherent in these ecosystems [1–4]. Modular food web theory, the study of low species food web configurations within a food web, seeks to identify how structure and interactions act together to mediate the stability of ecosystems [5,6]. The successes of this modular approach to understand the links between network structure, interaction strengths, and food web stability are encouraging. For example, modular approaches have found that weak interactions between trophic levels [3,7], coupling of strong and weak energy channels [8], and omnivory [9,10] can stabilize systems. Nonetheless, focusing on modules within the larger food web may not capture all network properties and, importantly for this study, tends to concentrate on species level food web structure at the cost of ignoring other biological structures that occur within species (for example, stage-structure); in doing so, important within-species structural mechanisms may be concealed [11].

The traits associated with the life-history stage of an organism can play a significant role in an organism’s physiological requirements and ecological interactions [12,13]. Moreover, life-history traits are influential factors in population [14,15] and community dynamics [16]. Therefore, the progression between life-history stages and divergence in life-history traits can have important implications for the dynamics of stage-structured food webs. For instance, mature organisms often consume prey at higher trophic positions than their juvenile counterparts due to differences in size or habitat [17]. These ontogenetic niche shifts can result in life-history omnivory or more specifically life-history intraguild predation (LHIGP) where the juvenile competes with the adult’s prey for resources [18–20]. Indeed, LHIGP modules have different dynamics over ranges of productivity than simple omnivory and intraguild predation (IGP) modules [18–22]. While it is known that there are dynamical implications of life-history structure for food web modules, how adding life-history will influence the link between food web structure, interaction strengths, and food web stability remains unresolved. This study seeks to explore how biomass allocation between life-history stages affects the stability of a LHIGP module.

We can draw information from unstructured modular theory to determine how stage-structure, through its effect on interaction strength, can mediate stability. Omnivory tends to stabilize unstructured food web modules through the re-distribution of biomass away from potentially strong interactions [10]. Components within an omnivory module that are strongly interacting tend to become oscillatory, and are stabilized by interactions with weakly interacting components in the module [7]. In line with unstructured models, it is possible that biomass movement due to reproduction and maturation may also mute potential oscillators within the LHIGP food web. Specifically, stage-structure may also act to redistribute energy (via ontogeny) away from a strong consumer-resource interaction and so stabilize the food web in a fundamentally similar way.

Historically, much of life-history theory was focused on how populations should manipulate their reproductive effort and age-at-maturation to mediate increases or decreases in abundance [14]. By extension, reproductive traits that reallocate energy between stages will be linked to community dynamics in stage-structured modules [23]. We seek to determine how diverting energy away from a strong interaction through changes in reproduction and maturation affects the local asymptotic stability (i.e. real part of the dominant eigenvalue of the system of equations [1]) in a module with LHIGP. This is done by investigating a simple model that allowed us to move from an unstructured food chain to a stage-structured LHIGP module. By starting with an unstructured food chain we were able to examine the potential for biomass reallocation between predator life-history stages in the stage-structured predator module to mute instabilities associated with the unstructured food chain [24].

## Model

### Model Development

A simple LHIGP module was adapted from the omnivory model by Vandermeer [25] by using ordinary differential equations for resources (*R*), consumers (*C*), and adding a stage-structured top predator with juvenile and adult stages (*P*_*J*_ and *P*_*A*_):

$$\frac{dR}{dt}=R\left(r(1-\frac{R}{K})-\frac{a_{CR}C}{1+b_{CR}R}-\frac{a_{PR}P_{J}}{1+b_{PR}R}\right)$$ (1)

$$\frac{dC}{dt}=C\left(\frac{a_{CR}R}{1+b_{CR}R}-\frac{a_{PC}P_{A}}{1+b_{PC}C}-d_{C}\right)$$ (2)

$$\frac{dP_{J}}{dt}=P_{A}\left(\frac{(1-s)a_{PC}C}{1+b_{PC}C}\right)+P_{J}\left(\frac{(1-m)a_{PR}R}{1+b_{PR}R}-d_{P}\right)$$ (3)

$$\frac{dP_{A}}{dt}=P_{A}\left(\frac{sa_{PC}C}{1+b_{PC}C}-d_{P}\right)+P_{J}\left(\frac{ma_{PR}R}{1+b_{PR}R}\right)$$ (4)

The resource, R (biomass (e.g. kg)), increases through time, t (e.g. month), following logistic growth with carrying capacity *K* (biomass) and growth rate *r*, and decreases through feeding by consumers, C (biomass), and juvenile predators, P_J_ (biomass), which follow a Type II functional response (functional response parameters *b*_*CR*_, and *b*_*PR*_). Following Vandermeer [25] *a*_*CR*_ and *a*_*PR*_ were considered consumption constants. A detailed description of the functional response parameters is provided in Vandermeer [25]. Resource consumption by consumers and juvenile predators increases their net biomass production. Juvenile predator biomass also increases through reproduction (*1-s*). Consumer biomass decreases through predation by adult predators, P_A_ (biomass), (Type II functional response parameters *a*_*PC*_, *b*_*PC*_) and background mortality (*d*_*C*_) and juvenile biomass decreases through maturation to the adult stage (*m*) and background mortality (*d*_*P*_). Adult predator biomass increases through consumption of consumer biomass (Type II functional response parameters *a*_*PC*_, *b*_*PC*_) and through maturation of juvenile predators, and decreases through reproduction (*1-s*) and background mortality (*d*_*P*_). The parameter (*1-m*) is the amount of energy remaining in the juvenile stage and *s* is the amount of energy remaining in the adult stage for somatic growth. The way that reproductive effort and age-at-maturation were incorporated here implies a negative correlation for reproductive effort and age-at-maturity with somatic growth (i.e. setting somatic growth equal to one minus the reproductive effort or maturation parameters); there are many examples where this trade-off exists in nature [26,27]. This module differs from other LHIGP modules previously used (e.g., [20,22]) in three main ways: we use logistic growth in the resource instead of semi-chemostat; values of *s* dictate the amount of energy that remains in the adult stage for somatic growth versus moving to the juvenile stage as reproduction instead of all the energy being used for reproduction (e.g., [20]) or net biomass after maintenance costs being allocated to reproduction (e.g., [22]); and finally the model represents only the unique case where the adult predator feeds on the consumer and the juvenile feeds on the resource (allowed to vary in [22]).

**Fig 1:**
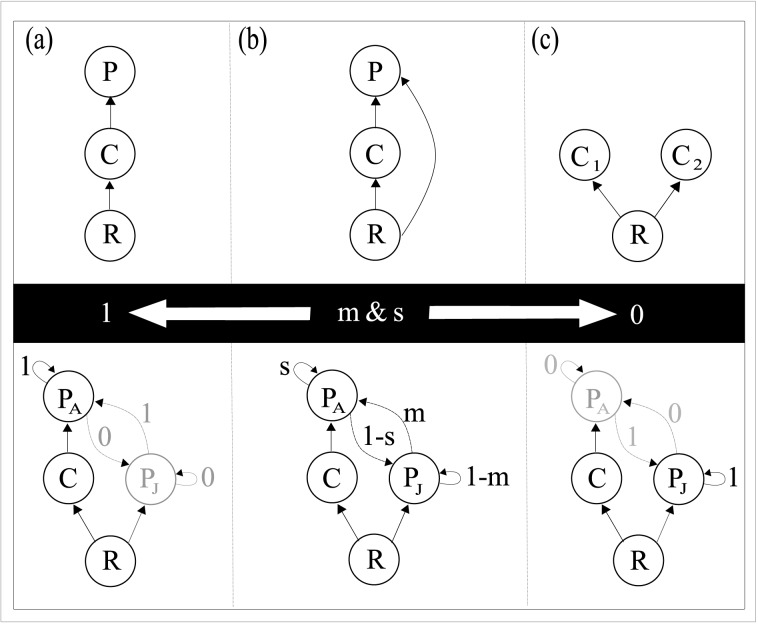
Nested stage-structured modules and their unstructured counterparts. The life-history intraguild predation (LHIGP) module when *m* and s are equal to 1 (a) is reduced to the straight chain food web module. Somewhere in between (b) the LHIGP module will have equivalent dynamics to the intraguild predation module; when *m* and *s* are equal to 0 (c) the LHIGP module is reduced to the competition module.

When *m* = 1 and *s* = 1, the module is reduced to the well-studied three species food chain module (Fig 1A). All of the energy gained by the adult predator is allocated towards reproduction and all of the energy in the juvenile stage is transferred to the adult stage essentially removing the juvenile stage. As *m* and *s* are decreased, competition between the predator and the consumer increases, and there will be a point where the module dynamics will be equal to the omnivory case (Fig 1B). And finally, when *m* = 0 and *s* = 0, the module is reduced to pure competition (Fig 1C). The parameters, *m* and *s* were set at the same value to be able to achieve these specific end points. When allowed to change independently, there are qualitatively similar results, with the value of *s* having a larger effect than *m* on the stability of the system as it controls the majority of the energy transfer, which is being diverted away from the stronger interaction (i.e. adult stage).

The end points represent populations that have either only an adult stage (food chain, m&s = 1) or a juvenile stage (competition, m&s = 0), while not biologically feasible, serve as representations of classical unstructured models. This nested model approach allows a clear examination on the role of energy transfer when adding stage-structure in the simplest way. This formulation allows us to move between nested modules that are both structured and unstructured, enabling us to distinguish the effect of altering the biomass allocation from other possible effects that would occur in more complicated models. Once the dynamics of this simple module are understood, complicated models that consider more realistic differences in feeding rates based on size and/or food-dependent maturation [18,19,22,28–30] should be examined to determine how this added detail affects the results.

## Equilibrium and Model Stability

Here, in all our numerical experiments we always start from the end case of a food chain (i.e., m = 1 and s = 1). We use previous results to inform our starting conditions (whether we are starting from an equilibrium or non-equilibrium attractor). We then vary one, and only one parameter of interest (the bifurcation parameter), and follow the fate of the interior equilibrium and its local stability (i.e., real part of the largest eigenvalue) and/or the local maxima and local minima on the attractor. This latter approach allows us to unfold the bifurcation parameter and follow non-equilibrium attractors as well, effectively determining the existence of the interior equilibrium at each new step of the bifurcation parameter, and then calculates the Jacobian of the full system [1] and the eigenvalues associated with this new parameter set. By continuity, we know that very small values of the modified parameter preserve the feasibility of the equilibrium solution. Thus, we follow the equilibrium (or non-equilibrium) solutions explicitly as we increase the parameter of interest.

The dominant eigenvalue of the system of equations (i.e., eigenvalue with the largest real part) determines the local stability of the system; when the largest real part is negative then all the eigenvalues are negative and the system is asymptotically stable (i.e. all nearby solutions will converge), when it is positive the system is unstable, and when it is zero the system is neutrally stable (i.e. solutions near zero will stay near zero, they will neither attract nor repel). The real part of the dominant eigenvalue also provides a measure of the relative local stability of the system, with the system reaching maximum stability where the real part of the dominant eigenvalue has its most negative value since the system is the furthest away from the Hopf bifurcation (i.e. the point where the system stability switches between positive and negative) [31]. Where the system was in a non-equilibrium zone (where the real part of the dominant eigenvalue was greater than zero), eigenvalues were supplemented with local minima and maxima values of the predator adult and juvenile stages, consumer, and resource biomass (i.e., points in the time series that display local minima or maxima). All results were obtained using numerical simulation models in Mathematica [32]. Only solutions with interior equilibriums (i.e., all state variables that are greater than zero) were recorded.

## Model Starting Conditions and Parameterization

In order to generally test whether biomass transfer between stages has the potential to alter the stability of a LHIGP module, we choose parameter combinations in our numerical experiments that result in the entire range of possible dynamic outcomes for the initial food chain endpoint (i.e., chaotic, cyclic, or stable). We then ask whether the initial source of instability affects the qualitative stabilizing effect of biomass transfer between stages. The basic food chain module is an appropriate starting point (Fig 1A) as the dynamics of the system are well known [24] allowing us to look at the general influence of LHIGP under different dynamical scenarios, in essence performing a sensitivity analysis over the entire suite of possible starting conditions. In what follows, we therefore separate the dynamics into four informative cases where the initial food chain is: stable (Case 1); cyclic, where the cycle is driven primarily by a strong consumer-resource interaction (Case 2); cyclic, where the cycle is driven primarily by a strong predator-consumer interaction (Case 3) and; complex dynamics where the cycles are an interaction of strong consumer-resource and strong predator-consumer interactions (Case 4).

**Fig 2:**
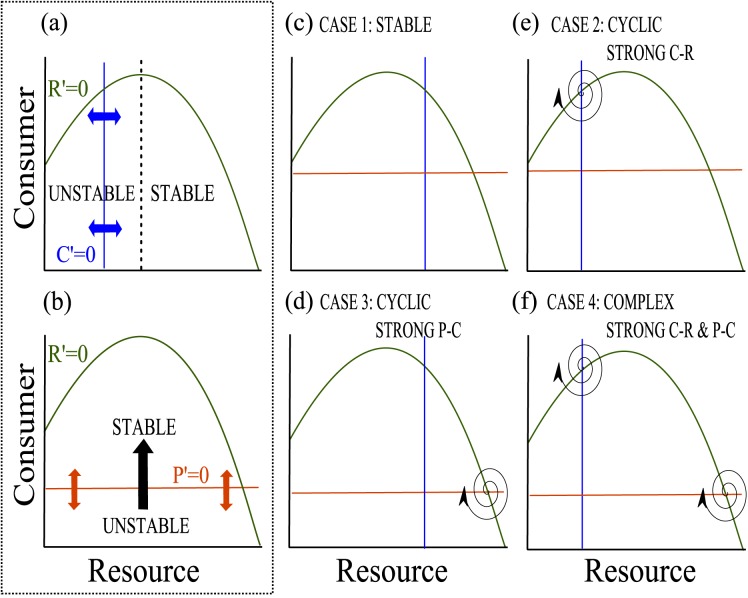
The predator, consumer, and resource isoclines of the food chain module. The location of the intersections of resource (R’), consumer (C’), and predator (P’) isoclines on reduced nullsurface of a food chain module, which is a projection of all zero isoclines onto the *C-R* plane, is directly related to the stability of the food chain. The C-R interaction is unstable when C’ lies to the left of the resource isocline hump and is stable when to the right of the hump (a). Similarly, when P’ is pushed from high to low C densities, the potential to drive P-C oscillations (depicted in this figure as an outwardly spiraling arrow) increases (b). All parameter combinations are represented in one of four basic cases: stable food chain (c), cyclic food chain where the cycle is driven primarily by a strong consumer-resource interaction (d), cyclic food chain where the cycle is driven primarily by a strong predator-consumer interaction (e) and, complex dynamics where the cycles are an interaction of strong consumer-resource and strong predator-consumer interactions (f).

Fortunately, previous food chain theory provides us with a method to clearly determine these 4 cases for the food chain starting point (*m* = *s* = 1) using a technique called reduced nullsurface analysis [24]. Fig 2 depicts the reduced nullsurface of the food chain, which is a projection of all isoclines that represent a growth rate of zero onto the *C-R* plane. This projection allows us to interpret the qualitative dynamics of the underlying parts of the food chain; in other words, whether the food chain can be expected to derive an oscillatory component from the *C-R* interaction, or the *P-C* interaction, or both. As is well known from *C-R* theory, the *C-R* interaction is unstable when the consumer isocline lies to the left of the resource isocline hump and becomes stable when to the right of the resource isocline hump (Fig 2A, P = 0). A similar, but less well known, result occurs for the predator in the *P-C* isocline [33]. In this case, when the predator isocline is pushed from high to low *C* densities by changing model parameters, the potential for *P-C* driven oscillations increases (Fig 2B). In this case, only attributes of the top predator are changed, so C’ and P’ change, however, R’ remains constant. In Fig 2, since all zero isoclines are projected onto the C-R plane, the P-C intersection is at the point where the predator, consumer, and resource isoclines meet, which on the C-R plane is represented where the predator and resource isoclines cross. This occurs because the P and R-isoclines are invariant across P (i.e., they do not change shape) and the consumer isocline deflects to the right as P increases, necessarily driving the interior equilibrium intersection at the precise point of P’ and R’ identified on the reduced nullsurface. With these results, McCann and Yodzis [24] showed that the bifurcation structure of the food chain was easily understood from the positions of the reduced nullsurface.

In summary, this simple result means that: (i) the C-R sub-system becomes an oscillator when the C-isocline is pushed up to low R densities (Fig 2A), and; (ii) the P-C sub-system becomes an oscillator when the P-isocline is pushed up to low C densities (Fig 2B). Additionally, when a P-C-R food chain is unstable, it can be attributable to (i) instability of P-R subsystem, (ii) instability of C-R subsystem or (iii) both. Given this mathematically and numerically proven result [24], we can arrange the C-isocline and P-isocline relative to the R-isocline to make the following 4 qualitatively interesting cases highlighted in Fig 2C–2F: All stable (C isocline to right of hump, or high R densities, P isocline at high C densities; Fig 2C); C-R oscillatory, P-C not oscillatory (C isocline to low densities, or left of R-isocline hump; P at high C densities; Fig 2E); C-R not oscillatory, P-C oscillatory (C isocline to right of hump, or high R densities, P isocline pushed to low C densities; Fig 2D); and C-R oscillatory; P-C oscillatory (C-isocline pushed to low R densities; P-isocline pushed to low C densities; Fig 2F)

For the LHIGP case, when the bifurcation parameter is non-zero (i.e. *m* = *s* ≠ 1) the reduced nullsurface is no longer useful, but the approach we adopt importantly allows us to determine where the oscillator is in the system and then ask how the stage-structure (unfolded by the *m* and *s* parameters) influences that oscillator (i.e. inhibits it or not).

The parameter values are the same for all four cases (*r* = 1.5, *K* = 1, *a*_*CR*_ = 3, *a*_*PC*_ = 1, *a*_*PR*_ = 0.2, *b*_*CR*_ = 1.5, *b*_*PC*_ = 1, *b*_*PR*_ = 1) except for predator and consumer mortality rates (*d*_*P*_ and *d*_*C*_), which were altered to change the relative interaction strengths: *d*_*C*_ = 0.3 (Cases 2 and 4), *d*_*C*_ = 0.5 (Cases 1 and 3), *d*_*P*_ = 0.1 (Cases 3 and 4), and *d*_*P*_ = 0.15 (Cases 1 and 2) (Table 1). By changing the mortality term we change the relative interaction strength; i.e. the strength of the coupling terms relative to the strength of the loss terms [34]. We could have just as easily changed the consumption constants yielding the same qualitative results. These particular parameter values were used to represent the four cases to test the influence of stage structure on all potential underlying attractors in the unstructured system. Based on the geometric explanation above, all parameter combinations that result in one of the four basic cases will result in qualitatively the same dynamics for the dominant real eigenvalue over the range of co-existence; this was verified in a sensitivity analysis using ten unique combinations of parameters representing the four cases; the response of the dominant real eigenvalue to changes in *m* and *s* was qualitatively the same with every parameter combination. We chose to examine these four cases, instead of a parameter combination for a specific system, in order to generally test the ability for the transfer of biomass between stages to alter the stability of the module representative of any LHIGP system. The consumption constant for the juvenile predator on the resource (*a*_*PR*_), which has no effect on the stability of the initial food chain, was varied to determine the effects of increasing the *P*_*J*_*-R* interaction strength on the stabilizing potential of diverting energy away from a strong adult predator-consumer interaction in the LHIGP module. The model experiment was repeated multiple times with a_PR_ varying from 0–1, recording the point where the maximum stability was reached.

**Table 1:** Values for predator and consumer mortality rates altered to change the relative interaction strengths to represent the four possible dynamic starting conditions.

Parameter	Case 1	Case 2	Case 3	Case 4
d_P_	0.15	0.15	0.1	0.1
d_C_	0.5	0.3	0.5	0.3

## Results

**Fig 3:**
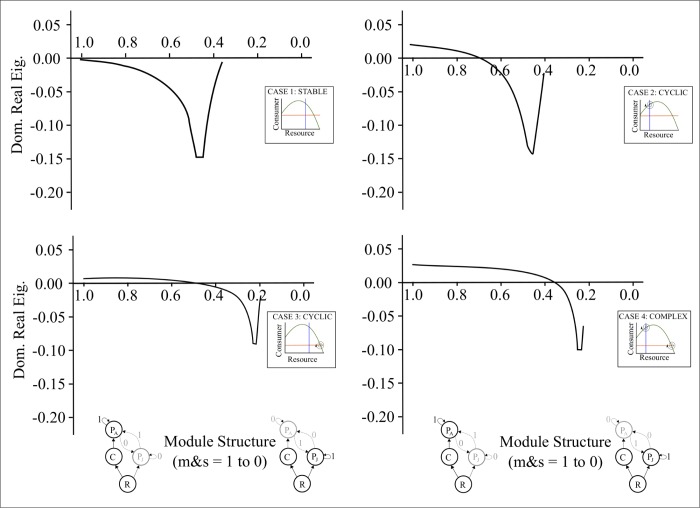
Eigenvalue stability in the LHIGP module. Patterns of stability for Cases 1–4 (a-d) starting from a food chain module and moving towards an exploitative competition module as *m* & *s* decrease from 1 to 0 (Parameter values: r = 1.5, K = 1, a_CR_ = 3, a_PC_ = 1, a_PR_ = 0.2, b_CR_ = 1.5, b_PC_ = 1, b_*PR*_ *= 1*, d_C_ = 0.3 (b and d), 0.5 (a and c), d_P_ = 0.1 (c and d), 0.15(a and b)).

**Fig 4:**
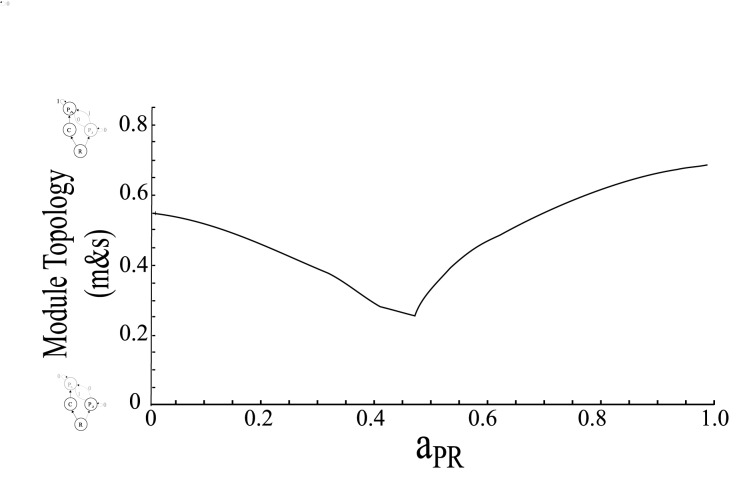
Maximum stability of the LHIGP module. Patterns of the maximum point of stability reached across nested food-web modules over a range of juvenile predator consumption constants for Case 1, which is representative of the qualitative patterns for all four cases (Parameter values: *r* = 1.5, K = 1, a_CR_ = 3, a_PC_ = 1, a_PR_ = 0–1, b_CR_ = 1.5, b_PC_ = 1, b_*PR*_ *= 1*, d_C_ = 0.5, d_P_ = 0.15).

In general we found that intermediate levels of LHIGP were stabilizing (Fig 3) regardless of the initial sources of instability (Cases 1–4). While initial sources of instability (Cases 1–4) did not have a large effect on the dynamics of the real part of the dominant eigenvalue in the LHIGP module, there were implications regarding where the maximum stability of the LHIGP module occurred in relation to the distribution of biomass (Fig 3). Finally, the point of maximum stability (Fig 4) was affected by the juvenile consumption constant (*a*_*PR*_).

Irrespective of the initial source of instability (Cases 1–4), the real part of the dominant eigenvalue of the system displayed a checkmark pattern as *m* and *s* decreased from 1 to 0 (Fig 3A–3D). For each case, the minima and maxima in the non-equilibrium zone (where the real part of the dominant eigenvalue was greater than zero) displayed decreasing cycles (i.e., reduced local maxima and increased local minima) to the point of the Hopf bifurcation (i.e. where the eigenvalue switched from positive to a negative), at which point the system is reduced to a single non-oscillatory attractor (Figures A-C in S1 File). The stable system and the system with an oscillatory *C-R* interaction (Cases 1 and 2, Fig 3B) in the initial food chain achieved higher maximum stability (i.e. the dominant real eigenvalue reached -0.15) with a maximum stability that occurred closer to food chain endpoint (i.e. *m* and *s* values between 0.4 and 0.6) than cases with strong predator-consumer interactions (i.e. *m* and *s* values between 0.2 and 0.4) (Case 3–4, Fig 3C and 3D). In all cases, as the system became more like the classic competition food web module, with most of the energy being allocated to the juvenile predator (as m and s approached 0), the predator was outcompeted by the consumer due to the low competitive ability of the juvenile predator (low a_PR_) and was no longer able to exist (Fig 3).

While it was necessary for *a*_*PR*_ to be weaker than *a*_*CR*_ for stability (not shown), consistent with most other studies ([18,19,22], see [35] for an opposing case), manipulating *a*_*PR*_ within this range affected the point of maximum stability in the system (Fig 4). For simplicity, only the maximum stability results from Case 1 are shown (Fig 4) as they are reflective of the qualitative patterns in all four cases. As *a*_*PR*_ increased, the point of maximum stability occurred at lower *m* and *s* values (closer to the classic competition module); then switched to increasingly higher *m* and *s* values (closer to the food chain module) at intermediate *a*_*PR*_ (Fig 4).

## Discussion

Recent work has argued that stage-structured food web theory may frequently contrast with classical food web theory [11]. This notion comes from the fact that since stage-structured models have some unique dynamical properties (e.g., cohort cycles [16]), it is unclear if a unique topology of community interactions will be stabilized in a manner equivalent to the instabilities associated with classical food web models. Given that specific differences in dynamical outcomes exist, it becomes important to ask whether stage-structure actually changes the general underlying mechanisms that drive stable webs. Much species-level theory, for example, has shown that diverting energy away from strong interactions towards weak to intermediate interactions can mute potentially strong interactions driving increased stability [7]. We showed that the transfer of energy through ontogeny to weak interactions can act as a powerful stabilizing agent in within-species models.

We investigated a simple model that allowed us to move from an unstructured food chain module to a stage-structured intraguild predation module, a well-studied system in both species-level and within-species theory, and finally to an unstructured competition module. When the initial chain was unstable due to a strong adult predator-consumer (*P*_*A*_*-C*) interaction (Cases 3 and 4 in results), diverting weak to moderate amounts of biomass into the juvenile predator from the strongly interacting adult predator resulted in stabilization. This stabilization lasts until the diverted biomass is large enough that most of the energy is allocated to the juvenile predator-resource interaction (*P*_*J*_*-R*) which is a weak competitor relative to the consumer-resource interaction, driving competitive exclusion of the predator. In summary, altering life-history in a manner that shunts weak to moderate amount of energy away from the potential adult predator oscillator acts as a potent stabilizer to the system. This result, whereby energy is deflected away from a potentially strong interaction, is analogous to the case where a new, weakly coupled, prey species is added to a system with a strongly interacting consumer and resource [7].

When the initial food chain is stable or the sole source of instability was a strong consumer-resource (*C-R*) interaction (Cases 1 and 2), the stability properties displayed the same checkmark pattern as biomass is diverted into the juvenile predator. This demonstrates that biomass reallocation between stages can also alter the effect of unstructured interactions on stability. Unlike in the cases with a strong adult predator oscillator (Cases 3 and 4), the reallocation of biomass between juvenile and adult stages affects the stability of the system by changing the amount of energy allocated to unstructured consumer-resource and competition interactions. At the food chain endpoint, high adult predator biomass dampens the *C-R* oscillator through a consumer-resource interaction reducing consumer biomass (*P*_*A*_*-C*) by diverting energy away from the strong *C-R* interaction. Increasing *m* and *s* reduces this dampening effect of consumer-resource interaction and increases the dampening effect of the competition interaction between the juvenile predator and the consumer. Stability increased at first with the addition of a weak *P*_*J*_*-R* interaction which increases competition for the resource, however, when the amount of energy being diverted to the juvenile stage increases to a point where the adult stage is no longer able to dampen the *C-R* oscillator the system becomes less stable. When there is a strong predator-consumer (*P*_*A*_*-C*) oscillator (Cases 3 and 4) the switch between being stable and unstable occurs closer to the unstructured competition endpoint than the food chain end point. Greater biomass reallocation between stages is required to directly stabilise the *P*_*A*_*-C* oscillator than to stabilise the *C-R* oscillator. It is well known in unstructured theory that diversity in feeding relationships across trophic levels acts to weaken interaction strengths (e.g., [36–38]). This study provides an example of stabilization through ontogenetic changes in feeding relationships, which is currently poorly understood [35].

The strength of the interaction between the juvenile predator and the resource had a strong effect on where maximum stability was reached in the LHIGP module (Fig 4). After intermediate levels of a_PR_, the strength of the juvenile predator–resource (*P*_*J*_*-R*) interaction was strong enough that the movement of biomass into the juvenile stage was no longer diverting energy away from a strong interaction into a weak interaction and was no longer stabilizing. This is consistent with research that found for coexistence to occur in the LHIGP module, the *P*_*J*_*-R* interaction needed to be a weaker interaction than the C-R interaction (e.g., [18,19,22]). This study shows, in addition, that within the range of coexistence, the strength of the interaction has important consequences for stability. Similarly, in an IGP module, how efficient the predator is at converting consumer and resource biomass into reproduction can determine the coexistence of the predator and the consumer [39]. If the indirect flow of energy through the consumer to the predator was higher than the direct flow from the resource to the predator, then the predator and the consumer could coexist [39]. Breaking down the consumption constant in this LHIGP module would allow us to determine the relative importance of conversion efficiency and attack rate in terms of stability in the region of coexistence. It is unclear, however, how differences between the conversion efficiencies of the adult and juvenile stages would affect the stability of the local attractor; a similar approach to that taken here could address this uncertainty.

We consider here the effect of increasing the flow of energy through ontogeny to a weak interaction (i.e. the juvenile predator) on the stability of the local attractor in order to follow food web theory that has generally examined the effect of interaction strength on the stability of local attractors. This was done at the risk of ignoring multiple basins. Other studies tend to look more broadly at bi-stability or persistence in LHIGP systems [20,40], seeking the range of parameters that yield coexistence. Under these conditions, researchers are able to contrast the importance of ecological interactions such as predation and competition for coexistence (e.g., [41,42]). Consistent with this research, we chose to include an ontogenetic specialist for the LHIGP predator, that has been shown to promote coexistence in a LHIGP module [22]. Future work should examine how these two different, but related, answers interact.

Only one type of structured module was used in this study to look for underlying stabilizing mechanisms, however, there is evidence from other modules that stabilisation through biomass allocation between stages could be pervasive in within-species modules. For example, cannibalism may stabilise consumer-resource dynamics in stage-structured modules by reducing predator population growth [21] in turn weakening the consumer-resource interaction. Again, as for within-species modules, weak to moderate cannibalism strengths would be stabilizing by diverting energy away from strong interactions, whereas strong cannibalistic interactions would initiate population instabilities [21]. Similarly, stage-structured refugia has been argued to weaken strong consumer-resource interactions by removing biomass from targeted prey stages weakening the interaction [43]. Collectively, these ideas suggest that the underlying mechanism of weak to intermediate interactions operating in classical food web theory may also be a general pattern operating in stage-structured theory; however, this study should be repeated using the above mentioned module formulations to be certain that the reallocation of biomass is the mechanism acting on stability.

While we have found a general congruence between classical food web theory and a simple food web extended to a stage-structured predator, more complicated models exist that we did not examine. Specifically, we manipulated set maturation and reproduction values, instead of including food-dependent maturation rates, which are known to yield dynamical properties that are unique to stage-structured interactions [16,44]. Additionally, stage-structured models often find alternative states where comparable classical models do not [29,45]. The presence of alternative states can be considered another form of instability since the presence of multiple attractors can cause state shifts, if the perturbation is large enough, reducing the relative stability of the any one attractor. By concentrating on the local stability of attractors we have ignored this important aspect of stability, however, alternative stable states may be more frequent in stage structured modules which adds a level of complexity that we have not examined in this study.

Nonetheless, while researchers have postulated that a stage-structured food web theory may deviate considerably from classical food web theory, we have shown that the underlying mechanism (i.e., weakening potentially strong and unstable interactions) also operates in simple stage-structured food web modules. While we were not able to test this in our current study, this result potentially occurs because cohort-driven (P_A_-P_J_) oscillations, like their C-R counterpart, occur from high growth rates or large consumption constants, and so any stable interaction that mutes the effect of these parameters, also mutes the destabilizing potential of the cohort-driven oscillator. In conclusion, where the flow of energy through species interactions mediates species-level module stability in classical food web theory, we argue that the flow of energy through biomass transfers in within-species modules has the potential to mediate stability in the same way. This general result suggests the existence of a powerful synthesis between population and food web ecology.

## Supporting Information

S1 File Minima and Maxima. Figures showing the minima and maxima for a) the resource, b) the consumer, c) the juvenile predator, and d) the adult predator starting from a food chain module and moving towards an exploitative competition module as maturation and somatic growth (*m* & *s*) decrease from 1 to 0 for Cases 2–4.
(DOCX)
