# Supplementary material for: Biomass Reallocation between Juveniles and Adults Mediates Food Web Stability by Distributing Energy Away from Strong Interactions
Source: PLoS One. 2017 Jan 23;12(1):e0170725. doi: 10.1371/journal.pone.0170725 (PMC5256945; doi:10.1371/journal.pone.0170725)
Supplement: S1 File — Figures showing the minima and maxima for a) the resource, b) the consumer, c) the juvenile predator, and d) the adult predator starting from a food chain module and moving towards an exploitative competition module as maturation and somatic growth (m & s) decrease from 1 to 0 for Cases 2–4. (DOCX) [file pone.0170725.s001.docx]

# Supporting Information

**S1 Fig A. Case 2 Minima and Maxima**. Minima and maxima for a) the resource, b) the consumer, c) the juvenile predator, and d) the adult predator starting from a food chain module and moving towards an exploitative competition module as maturation and somatic growth (*m* & *s*) decrease from 1 to 0 for the case starting from a life history intraguild predation (LHIGP) module with an unstable C-R interaction (Case 2) (Parameter values: r = 1.5, K = 1, a_CR_=3, a_PC_=1, a_PR_=0.2, b_CR_=1.5, b_PC_=1, b*_PR_=1,* d_C_=0.3, d_P_=0.15)

**S1 Fig B. Case 3 Minima and Maxima**. Minima and maxima for a) the resource, b) the consumer, c) the juvenile predator, and d) the adult predator starting from a food chain module and moving towards an exploitative competition module as maturation and somatic growth (*m* & *s*) decrease from 1 to .4 for the case starting from a life history intraguild predation (LHIGP) module with an unstable P-C interaction (Case 3) (Parameter values: r = 1.5, K = 1, a_CR_=3, a_PC_=1, a_PR_=0.2, b_CR_=1.5, b_PC_=1, b*_PR_=1,* d_C_=0.5, d_P_=0.1) .

**S1 Fig C. Case 4 Minima and Maxima**. Minima and maxima for a) the resource, b) the consumer, c) the juvenile predator, and d) the adult predator starting from a food chain module and moving towards an exploitative competition module as maturation and somatic growth (*m* & *s*) decrease from 1 to .4 for the case starting from a life history intraguild predation (LHIGP) module with unstable P-C and C-R interactions (Case 4) (Parameter values: r = 1.5, K = 1, a_CR_=3, a_PC_=1, a_PR_=0.2, b_CR_=1.5, b_PC_=1, b*_PR_=1,* d_C_=0.3, d_P_=0.1) .
